# The Influence of Growth and Maturation on Stretch-Shortening Cycle Function in Youth

**DOI:** 10.1007/s40279-017-0785-0

**Published:** 2017-09-12

**Authors:** John M. Radnor, Jon L. Oliver, Charlie M. Waugh, Gregory D. Myer, Isabel S. Moore, Rhodri S. Lloyd

**Affiliations:** 1 Youth Physical Development Centre, School of Sport, Cardiff Metropolitan University, Cyncoed Campus, Cyncoed Road, Cardiff, CF23 6XD UK; 2 Sport Performance Research Institute New Zealand, AUT University, Auckland, New Zealand; 3 Division of Sports Medicine, Cincinnati Children’s Hospital Medical Center, Cincinnati, OH USA; 4 Department of Pediatrics and Orthopaedic Surgery, College of Medicine, University of Cincinnati, Cincinnati, OH USA; 5 The Micheli Centre for Sports Injury Prevention, Boston, MA USA; 6 Department of Physical Therapy, University of British Columbia, Vancouver, BC Canada; 7 Centre for Sport Science and Human Performance, Waikato Institute of Technology, Waikato, New Zealand

## Abstract

Hopping, skipping, jumping and sprinting are common tasks in both active play and competitive sports. These movements utilise the stretch-shortening cycle (SSC), which is considered a naturally occurring muscle action for most forms of human locomotion. This muscle action results in more efficient movements and helps optimise relative force generated per motor unit recruited. Innate SSC development throughout childhood and adolescence enables children to increase power (jump higher and sprint faster) as they mature. Despite these improvements in physical performance, the underpinning mechanisms of SSC development during maturational years remain unclear. To the best of our knowledge, a comprehensive review of the potential structural and neuromuscular adaptations that underpin the SSC muscle action does not exist in the literature. Considering the importance of the SSC in human movement, it is imperative to understand how neural and structural adaptations throughout growth and maturation can influence this key muscle action. By understanding the factors that underpin functional SSC development, practitioners and clinicians will possess a better understanding of normal development processes, which will help differentiate between training-induced adaptations and those changes that occur naturally due to growth and maturation. Therefore, the focus of this article is to identify the potential underpinning mechanisms that drive development of SSC muscle action and to examine how SSC function is influenced by growth and maturation.

## Key Points

**Table:** 

Stretch-shortening cycle (SSC) performance increases with age in various forms of hopping, jumping, and sprinting tasks.
Research suggests that changes in the neuromuscular system during growth and maturation include increases in muscle size, pennation angle, fascicle length, tendon stiffness, motor unit recruitment and preactivation.
Combined, these adaptations may result in an improved SSC function due to increased elastic energy reutilisation, increased neural potentiation and an enhanced stretch-reflex contribution, predominantly due to an increase in force producing capabilities and a reduced electro-mechanical delay.

## Operational Definitions

- *Childhood* represents the developmental period of life from the end of infancy to the beginning of adolescence. The term children refers to girls and boys (generally up to the age of 11 and 13 years, respectively) who have not developed secondary sex characteristics.
- The term *adolescence* refers to a period of life between childhood and adulthood. Although adolescence is a more difficult period to define in terms of chronological age due to differential maturation rates [1], girls 12–18 years and boys 14–18 years are generally considered adolescents.
- The terms *youth* and *young athletes* represent global terms which include both children and adolescents.
- *Growth* refers to quantifiable changes in body composition, either the size of the body as a whole or the size of specific regions of the body [2].
- *Maturation* refers to qualitative system changes, both structural and functional, in the body’s progress toward adulthood, such as the change of cartilage to bone in the skeleton, appearance of pubic hair or menstruation [1, 2]. The timing and tempo of maturation varies greatly between individuals during growth. Timing refers to when specific maturational events occur and tempo refers to the rate at which maturation progresses. All tissues, organs and systems of the body mature with growth, but they do so at different times and rates [1]. Within the current article, maturation is referring to biological maturation unless specifically stated.
- *Natural development* represents the increase in physical ability (strength, power, speed, etc.) that is apparent in children as they experience growth and maturation, independent of any specific physical training.
- *Adaptation*, in the context of this article, refers to changes in structure or neuromuscular properties.
- *Peak height velocity* is a somatic biological maturity indicator and reflects the maximum acceleration of growth during adolescence, providing a universal landmark to reflect the occurrence of other body dimension velocities within and between individuals [3].

## Introduction

Active play constitutes a large part of physical activity for children [4], with various forms of hopping, skipping and jumping tasks being performed frequently during the early years. Additionally, sprinting, jumping and throwing are key components of athletic motor skill competencies that are important for success in most sports [5]. These movements utilise the stretch-shortening cycle (SSC), which is characterised by an eccentric ‘stretching’ action prior to a subsequent concentric ‘shortening’ action [6]. The prior eccentric stretching (e.g. pre-load of the muscle) has been shown to enhance the performance of the final concentric phase in comparison to an isolated concentric action [7, 8]. For example, jump height improves 18–30% in adults when utilising a preceding countermovement [9, 10]. These values may be lower in a younger population, however, as pre-stretch only increased jump height in children by approximately 1–5% [11]. The SSC has been categorised into fast and slow actions based on a ground contact thresholds [12]; ground contact times shorter than 250 ms were classified as fast SSC activities, whereas slow SSC actions have ground contact times in excess of 250 ms. It has been suggested that slow SSC actions may enable greater force production because of increased contraction time and working range [7, 13], whereas fast SSC actions promote greater movement speed via elastic energy usage, stretch reflex contributions and a greater level of neural excitation from the preceding stretch [7, 13–17].

**Fig. 1:**
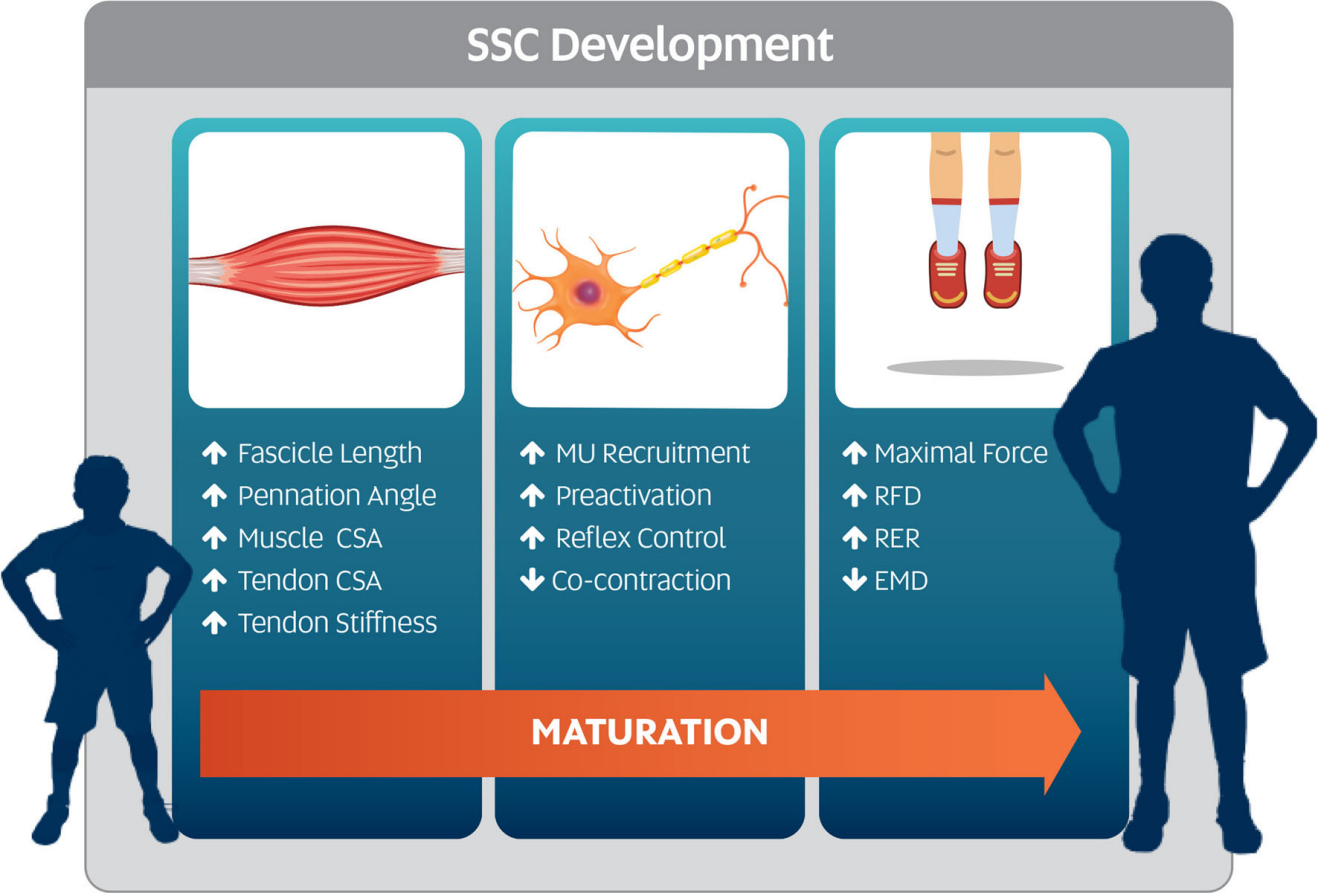
Visual representation of the primary mechanisms underpinning growth- and maturity-related changes in stretch-shortening cycle function. *CSA* cross-sectional area, *EMD* electromagnetic delay, *MU* motor-unit, *RFD* rate of force development, *SSC* stretch-shortening cycle, *RER* rate of EMG rise

SSC performance increases non-linearly with age in various forms of hopping and jumping tasks [1, 18, 19], and the underlying mechanisms of such neuromuscular developments associated with maturation remain unclear. SSC performance is governed by effective neuromuscular function, requiring an efficient interaction between both neural and muscular systems [6, 8] and the structure of the musculo-tendon unit (MTU) [20, 21]. Figure 1 presents a visual representation of the primary neuromuscular and structural qualities that are likely to influence the natural development of SSC function. Specifically, age-related developments in the neural system that could influence SSC function include greater preactivation [22, 23], increased stretch reflex magnitude [24] and musculotendinous stiffness [25], and reduced co-contraction ratios [26]. A number of musculo-tendinous adaptations also occur throughout childhood and adolescence, including increases in muscle thickness, cross-sectional area (CSA) and fascicle length, and changes in fascicle pennation angle (Table 1) [27–29]. Furthermore, adaptations to the tendinous structures occur, including increases in tendon CSA, length and stiffness [28, 30–34]. This review explains how the qualities highlighted in Fig. 1 develop throughout maturation, and the manner in which development influences SSC function in children and adolescents.

**Table 1:** Influence of structural and neural adaptations throughout biological maturation on stretch-shortening cycle function

Adaptation with maturation?	Influence on kinetic variables	Likely influence on stretch–shortening cycle function	Supporting evidence
Fibre type composition	Force production Shortening velocity	Increased force production Increased RFD	[33–36]
Increased muscle size	Force production	Increased impulse Increased RFD Increased cross bridges Increased EE storage	[5, 25, 26, 37, 39, 40, 56, 59, 60]
Increased pennation angle	L-T and F-V relationship *Gearing*	Increased force production Increased RFD Increased EE reutilisation Increased stiffness	[27, 52, 55, 60, 64, 65, 70, 73–78]
Increased fascicle length	Shortening velocity	Increased RFD Increased EE reutilisation Increased stiffness	[25, 26, 32, 37, 60, 65, 68, 70, 72]
Increased tendon size	Rate of force production	Increased tendon stiffness Increased RFD	[25, 26, 28, 29, 31, 84]
Increased tendon stiffness	Rate of force production	Increased RFD Decreased EMD Increased stretch reflex	[17, 23, 26, 28, 29, 32, 92, 94, 99–101]
MU recruitment	Force production	Increased RFD Increased contraction speed	[33, 42, 103–107]
Co-contraction	Force production	Increased EE reutilisation Increased stretch reflex Increased neural potentiation	[4, 17, 23, 42, 108, 109, 111]
Preactivation	Rate of force production	Reduced EMD Increased RFD	[20, 21, 114, 115]
Reflex control	Force production	Increased force production Increased RFD Increased stiffness	[20–23, 60, 72, 114]
RER	Rate of force production	Increased RFD	[33, 41, 94, 107, 118]

*EE* elastic energy, *EMD* electromagnetic delay, *F-V* force–velocity, *L-T* length–tension, *MU* motor-unit, *RER* rate of EMG rise, *RFD* rate of force development

## Structural Adaptations

### Fibre Type Composition

Differences in fibre type composition between children and adults has been proposed as one of the reasons that children produce less force than adults [35]. Research surrounding the effects of age on fibre type composition is scarce, and researchers have suggested ethical constraints as the main reason behind this [36]. From the available research into fibre type composition between adults and children, there is no clear consensus as to whether these differences exist. Children aged 3–21 months have been shown to have a lower percentage of type IIb fibres in comparison to adults (6.2 ± 1.1% [mean ± standard deviation (SD)] vs. 20.5 ± 1.6%), and a higher proportion of type IIa and type I fibres. Furthermore, a previous study has reported that the proportion of type I fibres decreases from approximately 65% at age 5 years to 50% at age 20 years [36]. Longitudinal data have demonstrated that gender differences in fibre type composition may also become apparent as adolescents transition towards adulthood. Research has shown that type I fibre percentage tended to increase in women (51 ± 9% to 55 ± 12%) and decrease significantly in the men (55 ± 12% to 48 ± 13%) between the ages of 16 and 27 years. On the other hand, there has been support for similar fibre type composition between children and adults [37].

While the available data are conflicting, the majority of research suggests that there may be child–adult differences in fibre type composition. Considering the fact that type I fibres produce less force and have a slower shortening velocity than type II fibres [38], throughout maturation the potential higher proportion of type II muscle fibres may result in an improved ability to rapidly produce force, resulting in greater benefit from the SSC.

### Muscle Size

During growth, large increases in muscle size are seen in a number of lower limb muscles [27, 28, 39]. Adults demonstrate greater muscle thickness than children [33, 39] and older adolescents exhibit greater muscle thickness relative to their younger peers [28, 32]. A recent review reported that the contribution of hyperplasia to changes in human muscle CSA is assumed to be small [40], and therefore the developmental increases in muscle size could mainly be attributed to increases in fibre hypertrophy. Considering that the muscle’s anatomical CSA (ACSA) is a major predictor of maximum strength and power in both adults and children [41, 42], increases in muscle size are a major factor contributing to the improved capacity to produce force as children experience biological maturation, resulting in greater performance outcomes (e.g. jump heights) during SSC activities.

Maximal muscle force is lower in children than in adults [30, 43, 44], but when normalised to body mass or muscle size, child–adult and sex-related differences in relative force production are inconclusive. There is evidence indicating that adults have a greater muscle force when normalised to body mass [25, 43, 45] and ACSA [25, 43, 44]; however, the majority of muscles studied have been pennate in nature [27, 32, 34, 46–56], and ACSA does not account for the entire contractile mass in a pennate muscle. Conversely, physiological CSA (PCSA) includes all sarcomeres in parallel and theoretically represents the sum of the CSAs of all the muscle fibres within the muscle [57]. PCSA can be calculated as the ratio between muscle volume and optimum fascicle length [27]. When normalising force to PCSA, boys were found to have a 21% higher force per unit area of muscle than men, also known as muscle-specific tension [56]. However, a number of methodological assumptions and omissions were made during the research process, including the assumption that the voluntary activation of the muscle was 100% in both groups, which have since been addressed in a follow-up study that identified no difference in muscle-specific tension between children and adults [39]. This finding indicates that increased muscle strength during growth could be less associated with muscle quality, although this is the only study to report such findings, and thus knowledge of child–adult differences in muscle-specific tension is still lacking.

As muscle size increases during growth and biological maturation [27, 28, 39], higher force output may be achievable during both the concentric and eccentric phases of the SSC. While this is yet to be researched, in isolated concentric and eccentric muscle actions, muscle size has been correlated with both quadriceps and hamstring concentric strength and hamstring eccentric strength [58]. The benefit of increased concentric strength during SSC actions include greater impulse, defined as the product of force and the period of time in which the force is expressed [59] and rate of force development (RFD), and therefore a superior performance during sprinting and jumping tasks [60, 61]. However, the benefits of increased eccentric strength are less clear. During a SSC action, the force produced is heavily dependent on the conditions involved in the eccentric phase [62]. Enhanced force generation during the eccentric phase may result in a greater number of active actin–myosin cross bridges, thus increasing the potential for contractile potentiation during the stretch [62]. Additionally, as muscles increase in size during growth, the higher forces during the eccentric phase may result in an increased potential for storage of elastic energy [8]. More force produced during the eccentric phase may result in the muscle resisting stretch, causing a greater tendinous lengthening [8, 62], and resulting in a more efficient elastic energy storage and reutilisation due to the shorter amortisation periods [63]. The influence of muscle size on the eccentric and concentric phases of the SSC has not been quantified and still needs further research before we can understand how muscle size may influence the underpinning mechanisms of SSC actions. Voluntary strength increases by a larger extent than anthropometric measures in pre-pubertal children [44], indicating that muscle strength depends not only on the muscle mass but also on the extent to which it is activated. This suggests that there are additional structural and neuromuscular qualities that develop throughout biological maturation that drive increased force production as children transition to adults.

### Muscle Architecture

Different muscles have distinct functions about each articulation, with each muscle group being of a specific design for their optimised performance [64]. The architectural arrangement of fibres within the muscle is important because it has implications for muscle function [65], particularly the fascicle’s force–length and force–velocity characteristics [66, 67]. There is substantial variability in measurements between distal, central and proximal aspects of a muscle that reflect the heterogeneity of pennate muscles, making direct comparisons between specific muscle groups difficult [27]. Whilst increases in muscle CSA directly correlate with force output increases during growth, changes in the specific architecture of the muscle may play a large role in strength gains as children transition towards adulthood. Despite these conceptions, there is a surprising dearth of evidence that has specifically investigated alterations in muscle architecture as a result of growth and development.

#### Fascicle Length

Fascicle length likely impacts muscle function by influencing the shortening velocity of a muscle. Longer fascicles result in an improved ability to produce force at higher velocities and over larger length ranges [68]. While there is limited research in human subjects, the relationship between fascicle length and shortening velocity has been investigated in animal models. Analysis of the feline semitendinosus demonstrated that the longer fascicles exhibited by the distal head resulted in a significantly faster shortening velocity than the proximal head [69]. In human subjects, trained sprinters have longer muscle fascicles than endurance runners [70], which highlight the importance of fascicle length for shortening velocity and explosive force production.

Fascicle length of the lower limb muscles are usually longer in men and women in comparison to boys and girls, respectively [27, 28, 34, 39]. Furthermore, 15-year-old adolescents have significantly longer muscle fascicles than children, but do not differ from adults [28]. This may imply that fascicle length reaches adult levels by the approximate age of 15 years; however, more research is needed before any substantial claims are made about the growth rates of tendon structures throughout adolescence. As longer fascicles allow for greater absolute maximum shortening velocities, these findings help explain significantly slower contraction velocities in boys compared with men [71]. Interestingly, involuntary muscle action responses, measured via supramaximal electrical stimulations, do not appear to change throughout maturation [72], which suggests that increases in fascicle length during this period would allow for the production of higher forces at similar contraction speeds, likely a result of more in-series contractile elements working simultaneously [68]. Nevertheless, longer fascicles should have a positive influence on the RFD. Gastrocnemius fascicle length was found to be a positive predictor of RFD during a countermovement jump in adults [73] and may highlight the potential for improved SSC function throughout childhood and adolescence, should greater fascicle lengths increase their ability to develop force rapidly. Moreover, the ability to develop force rapidly may ultimately enhance SSC performance by reducing ground contact times, leading to an improved mechanical efficiency by the reutilisation of elastic energy [63]. Additionally, shorter contact times could influence the impact neural potentiation has on the subsequent concentric action, as longer transition times between the eccentric and concentric contraction causes a decay in the magnitude of potentiation [74]. Increased RFD may also reduce yielding upon ground contact, enabling a greater overall system stiffness, which has established links with SSC-related jumping and sprinting performance [75].

#### Pennation Angle

Alterations in pennation angle throughout biological maturation appear to be muscle and site specific. The pennation angle of the knee extensor muscles seems to remain consistent from childhood through to adulthood [27, 28], whereas the pennation angle of the gastrocnemius medialis has been reported to increase from birth before becoming stable following the adolescent growth spurt [29, 76, 77], which might suggest that relative muscle growth to bone growth underlies differences in pennation adaptions in given limb segments. An increase in pennation angle throughout maturation might be expected to improve the force-generating capabilities of a muscle [57], and therefore improve the function of the SSC. For a given muscle volume, a larger pennation angle will increase the PCSA [78], which would result in a greater number of contractile elements attaching to the aponeurosis or tendon for a greater force transfer [79].

Fascicle pennation not only influences strength by enabling a greater PCSA, but it is functionally important as an increase in pennation (in conjunction with muscle thickening) during a contraction means that fascicles do not need to shorten as much overall, permitting slower fascicle velocities in relation to whole muscle or MTU velocities in a process known as *gearing* [67]. By enabling the muscle to (a) operate on a more optimal region of its force–velocity curve and (b) work at a favourable region of its force–length curve over a longer period, this maximises the force that the muscle can develop, without impacting on the capacity for rapid movement production [80]. Intuitively, a greater resting pennation would result in a higher *gearing* ratio, facilitating the muscle to take advantage of the force–velocity relationship during the SSC action. This would allow an individual to produce either more force at the same velocities, or greater velocities at similar forces.

Finally, a larger pennation angle may result in a greater passive resistance and therefore increased stiffness during SSC activities [73, 81]. Subjects with highly pennate muscles have greater early RFD during drop jumps, attributed to having an enhanced ability to cope with eccentric loads [54]. It has been hypothesized that because of the indirect line of pull of pennate muscles, a highly pennate muscle will have an increased ability to resist external forces [73], as the direct tendon force acting on the muscle are dissipated along the aponeurosis. An increased stiffness upon landing could lead to shorter ground contact times and therefore a better reutilisation of elastic energy [63].

Consequently, increases in pennation angle throughout maturation results in superior SSC performance through greater *gearing* and more force due to exploitation of both the force–velocity and length–tension relationships, in addition to the greater number of muscle fibres inserting to the aponeurosis [68], resulting in greater force being transferred to the skeleton. Additionally, a greater ability to cope with the high eccentric loads during SSC activities may result in greater RFD in these specific muscle actions [54].

### Tendon

Tendons are interposed between muscles and bones to form an MTU that transmits muscular forces directly to the bone, creating movement or stability around a joint. Tendons are predominantly composed of collagen fibrils which are arranged in a number of hierarchical structures [82] and oriented with the line of force transmission [83]. With the progression in technology, researchers have a better understanding of the three-dimensional fibriller structure of the tendon, which also includes horizontally and transversely oriented collagen fibrils forming spirals and plaits [84]. This complex biological structure and the fact that tendons are fibroelastic in nature, permits tendons to withstand heavy forces whilst maintaining its structural integrity, allowing the transfer of force between the muscle and bone to occur with minimal dispersion of energy [85]. The tendon plays a key role in SSC function, and the action of the MTU as a whole will differ from what happens to the muscle fascicles and tendon individually, influencing both force output and economy [86].

#### Tendon Dimensional and Material Properties

Tendon development throughout childhood and adolescence involves both dimensional and material adaptations [28, 30, 31, 33]. The specific dimensions of the tendon has a large influence on its function. For example, long, thin tendons are more compliant [87] and can be described as force amplifiers, which take advantage of the tendon’s ability to store and reuse elastic energy (the amount of energy stored is directly proportional to its extension). In contrast, short, thick tendons are stiffer [87] and are more effective at transferring muscular forces to bone due to their resistance to being stretched, hence their association with greater RFD and reduced electro-mechanical delay (EMD; the delay between muscle activation and the onset of force production [97]). Based on Hookean law, thicker tendons (greater CSA) are associated with higher stiffness as more spring-like material is arranged in parallel, whereas longer tendons are associated with lower stiffness as more spring-like tissue is arranged in series [88]. Therefore, the specific dimensions of the tendon could either result in a more economical SSC action (due to reutilisation of the elastic energy and the muscle having to perform less work), or increase speed of movement due to a faster transference of force from the working muscle to the bone.

Patella tendon length is significantly shorter in elementary school boys (~11 years) than in junior school boys (~14 years) and adult males, whilst patellar tendon CSA increased in size across all age groups [33], indicating a difference in the temporal growth of the tendon dimensions. However, both the length and CSA of the Achilles tendon were similar between the 14-year-olds and adults, and significantly greater than 11-year-olds, suggesting that these variables may become stable in boys around 14 years of age [32]. A dimensional bias underpinning the age-related increases in Achilles tendon stiffness between pre-pubertal children and adults evidences this reported effect [30]. Specifically, both tendon length and CSA were shown to increase by ~53 and ~93%, respectively, between 5- to 7-year-old children and adults, suggesting that the greater increase in CSA as opposed to tendon length would result in an increase in tendon stiffness [30]. Based on these data, tendon hypertrophy is likely a major adaptation influencing tendon stiffness and possible consequence of chronic loading through increased body mass and force production with age.

The internal properties of the tendon also play a role in stiffness properties. Young’s modulus is a dimensionless measure of a material’s stiffness and provides an indication of the underlying microstructure. It has been shown to increase with age [27, 28, 30, 33] and these internal adaptations play a role in the increased tendon stiffness reported throughout maturation. Increases in Young’s modulus are due to increases in collagen fibril diameter and density [89], alongside greater intra-fibrillar cross-linking [90], that are driven by the increases in tendon loading throughout maturation.

#### Tendon Stiffness

A tendon’s stiffness describes its resistance to elongation when a muscular force is applied. Stiffness of the patellar and Achilles tendon, and vastus lateralis aponeurosis have been shown to be stiffer in adults than in children [28, 30, 31, 34]. Furthermore, the stiffness of both the patellar and Achilles tendons appear to increase throughout childhood and into adolescence, with lower stiffness values reported in ~10-year-old children than in ~13-year-old children and adults [32, 33]. By age 15 years, the mechanical properties of the knee extensor tendon is similar to adults [28], suggesting that tendon stiffness may reach adult values following the approximate age of peak height velocity (PHV). PHV is a somatic biological maturity indicator, and reflects the maximum acceleration of growth during adolescence, providing a universal landmark to reflect the occurrence of other body dimension velocities within and between individuals [3]. These findings also suggest that differences in SSC ability between adolescent and adult populations may not be a result of tendon stiffness differences. Considering that the tendon is the optimal storage site for elastic energy, the key factor in the overall stiffness of the MTU is that the contractile element is required to be stiffer than the tendon to utilise its elastic storage potential. If this is not the case, the muscle could yield under load instead of the tendon, resulting in a sub-optimal muscle mechanics to produce force. Therefore, it could be postulated that adolescents do not have the ability to create this optimal muscle stiffness through the correct muscle activation strategies or motor unit recruitment [35].

Increases in body and muscle mass with growth and development result in an increased loading of the tendons [1]. Combined, body mass and force production has been shown to account for up to 78% of tendon stiffness variation in children and adults [30], demonstrating that age-related increases in tendon stiffness are likely attributable to increased tendon loading from weight-bearing tasks and increased plantar-flexor force production capabilities. This additional tendon loading with increases in body mass and force-producing capabilities as children mature likely acts as a stimulus for adaptation, resulting in changes to both tendon dimensions and material properties, which determine stiffness. Therefore, the observed increases in tendon stiffness during childhood and adolescence would appear to be mediated by growth- and maturity-related alterations of the material properties of the MTU [30, 31].

Gender differences in tendon stiffness have revealed inconclusive results. Recent research has demonstrated that males have a greater level of stiffness than females in both the patellar [91, 92] and Achilles tendon [93]. Previous research has suggested that gender differences may partly be due to differences in tendon dimensions [91]. Prior to adjusting for dimensions, tendon stiffness has been shown to be 115% greater in males than in females [91], whereas after adjusting for tendon size and CSA (Young’s modulus), the difference between males and females decreased to 53% [91]. There is, however, evidence to suggest that there are no differences in tendon stiffness between boys and girls, and women and men [31]; these authors suggested that the contrast in findings may be due to methodological differences when quantifying tendon stiffness. Interestingly, while no differences were identified in absolute tendon stiffness between adult males and females [31], the mechanisms underpinning tendon stiffness increases between childhood and adulthood may differ between sexes [31]. In males, increased stiffness appears to be modulated by the material properties of the tendon (i.e. Young’s modulus, a dimensionless measure of a materials stiffness), as the relative increase in tendon length and CSA have been shown to be approximately equal [31]. This would result in these two dimensions negating one another, demonstrating that the alterations to tendon dimensions would not affect stiffness. However, in adult females, tendon CSA increased by a greater amount than tendon length [31], suggesting that natural adaptation in tendon stiffness in females is due to both tendon hypertrophic responses and increases in Young’s modulus. These potential sex differences in the mechanisms of tendon development have not been reported in other studies, but provide an interesting rationale to explore the interacting effects of sex and maturation on mechanical properties of the tendon. Before making conclusive statements about gender differences in tendon stiffness, further research is needed in this area.

Tendon stiffness may affect functional movement by enhancing efficient transfer of force to the skeleton [94] influencing the RFD, an important determinant of force production characteristics [95]. A number of studies have reported that children have a reduced RFD relative to adults [43, 71, 72], which may partly be explained by the lower stiffness of the tendinous structures [96]. Additionally, EMD has been shown to influence the rapid generation of muscular force [98–100]. Children exhibit a longer EMD than adults [43, 71, 72] and EMD decreases as a child’s neuromuscular system matures [96]. Moreover, tendon stiffness is negatively correlated with EMD in children [96] and could help explain improvements in rapid force production ability as children age; certainly, such enhancements should improve SSC function with maturation. In contrast, in adults, a more compliant tendon has been shown to have a greater ability to store and reutilise elastic energy under the same loading conditions [101–103]. Throughout maturation, while tendon stiffness increases, tendon strain (a tendon’s displacement with respect to its resting length) does not change between childhood and adulthood [30, 33], and the increase in stiffness with maturation is likely due to the ability of the tendon to withstand greater force production, rather than reducing the displacement of the tendon [30, 33]. Conceptually, reutilisation of elastic energy and tendon stiffness may develop independently of each other; however, this has not been established, and further research is needed to examine changes in tendon properties and their influence on elastic energy reutilisation during maturation. Additionally, a stiffer MTU has been shown to elicit a greater stretch reflex in children [25], which would lead to shorter breaking phases and reduced contact times as well as greater electromyographic (EMG) activity [23].

## Neuromuscular Adaptations as a Result of Growth and Maturation

While the mechanical properties of the muscle and tendon will have a significant role in the regulation of the SSC, it is the interaction of the muscular and neural system that governs SSC performance [6, 8]. Similar to alterations in MTU structure, neural adaptations throughout childhood and adolescence will have a significant impact on the ability to regulate the SSC [22]. For example, a reduced RFD in children is partly caused by a greater agonist–antagonist co-contraction [25, 44], reduced ability to recruit high-threshold type II motor units [43, 71, 104] and lower muscle activation rates [43, 44], highlighting the importance of the neuromuscular system for explosive force production during SSC [35].

### Motor Unit Recruitment

The disparity in strength between children and adults, even when normalized to body size, has been attributed to children’s inability to activate their muscles to the same extent as adults [35]. In fact, children recruit a smaller percentage of their total motor-unit pool than adults during voluntary contractions [44, 105]. Using twitch interpolation techniques during maximal voluntary efforts to gauge the percentage of activated muscle, lower motor-unit activation was found in boys than in men (78 vs. 95%) during knee extension exercise [106]. Furthermore, motor-unit activation deficits seem to decrease with age [44, 107]. Considering that high threshold type II motor units have a larger twitch force, faster contraction speeds and rapid conduction velocities [108], researchers have hypothesised that it is a specific inability to recruit or utilise these motor units that limit a child’s force production capabilities [104]. Recently, it has been reported that boys have a higher EMG threshold, suggesting a delayed and lesser utilisation of type II motor units in progressive exercise compared with men [109]. Considering the difference between a child’s and adult’s ability to recruit high threshold motor units, it is postulated that children become more adept at recruiting high-threshold motor units as their central nervous system matures, which will lead to improvements in their ability to produce rapid force during SSC activities.

### Co-Contraction

Co-contraction is the simultaneous contraction of agonist and antagonist muscles around a joint [26] and provides joint stability. Children demonstrate greater co-contraction than adults [25, 44], which decreases with age [110]. Surface EMG analysis of the tibialis anterior and triceps surae musculature during quick-release movements at different levels of sub-maximal contractions revealed that co-contraction of the tibialis anterior was greater in younger participants [25]. Co-contraction can help provide joint stability, but greater antagonistic co-contraction will also increase the agonistic muscle energy cost of exercise [111] and reduce net force output [1]. Aberrant co-contraction neural factors likely result in less efficient movement and proprioception. When the magnitude or velocity of muscular contraction during SSC activities exposes the MTU to excessive tensile forces or rapid changes in length, the Golgi tendon organs increase afferent activity, thereby inhibiting the motor neurons innervating the agonist muscle and facilitating the antagonist motor units [112], reducing the overall efficiency of the SSC action by increasing ground contact times and reducing force output. A greater density and size of Golgi tendon organs is noted in children than in adults [113], but these undergo a process of desensitisation during maturation. Therefore, the process of reduced co-contraction throughout maturation will decrease agonist inhibition, resulting in a more efficient SSC action, as net force around each force would be higher. Reduced co-contraction may enable an increased pre-stretch of the muscle during the eccentric phase of the SSC [113], which would have positive effects on elastic energy reutilisation, stretch reflex response and neural potentiation [7].

### Preactivation

Preactivation is used to describe levels of muscle activity prior to an impact or landing [114]. This neuromuscular strategy has predominantly been measured during bilateral hopping tasks and has been defined as the muscle activation in the 50–100 ms prior to ground contact [115]. When performing two-legged hopping at a slow frequency, boys and men display similar muscle activation strategies; however, children display significantly lower preactivation at faster frequencies [116]. Similar results were reported during a 20 cm drop jump task, with children displaying significantly less preactivation than adults [23]. This reduced ability to utilise feed-forward mechanisms results in children producing longer ground contact times during hopping [116], which results in sub-optimal SSC function. Research shows that these neural mechanisms adapt with age, demonstrated by the fact that 15-year-old children produce significantly greater levels of pre-activity than 9 and 12 year olds during maximal hopping [22]. This trend could be stimulated by the greater drop heights and landing velocities that are evident in older children during hopping tasks. Due to the association between increased preactivation and increased muscle stiffness [117], the reduced feed-forward activity in younger children reflects a protective mechanism, aiming to prevent excessive rapid overload of the MTU upon ground contact. During maturation, SSC performance may improve as children transition from a reactive regulation of movement to a more pre-active control of movement, reflected by an increased reliance on preactivation prior to ground contact [22].

A greater muscle activation prior to ground contact may reduce EMD, as force can be immediately generated upon ground contact. However, the relationships between preactivation, EMD and the subsequent RFD during dynamic activity have yet to be established and further research is required to identify how muscle activation strategies could influence rapid force production in children. In addition, greater background muscle activity and short latency reflexes would dictate increased muscle activity during the eccentric phase of SSC, which will augment the interaction effects of the contractile and elastic elements and the storage and utilisation of elastic energy by reducing fascicle stretch and promoting length change in the tendinous structures.

### Reflex Control

The mean EMG from 30 to 60 ms, 60 to 90 ms and 90 to 120 ms after landing or impact is used to represent short-, medium- and long-latency stretch reflex components, respectively [114]. The short-latency reflex reflects the spinal involuntary command to activate the muscle during the 30–60 ms time phase of ground contact [115], whilst the medium- and long-latency reflex allows for supraspinal input [114]. Stretch reflex activity has been shown to improve with age in pre-pubertal boys and girls [24]. However, the researchers used protocols that involved isolated joint actions and an artificial stimulus to invoke the twitch response, which fails to elicit sufficient tension modifications to activate Golgi tendon organs and bypasses muscle spindle activation [116]. When using a SSC task to quantify the stretch reflex, researchers have found that the reflex amplitude is reduced in children compared with adults during a drop jump task [23]. Additionally, children have a greater reliance on longer-latency stretch reflexes during repeated hopping-in-place [22, 116]; however, as they transition through to adolescence, they appear to become more adept at regulating lower-limb stiffness through a greater utilisation of short-latency stretch reflexes [22]. Improved spindle sensitivity, maturation of the sensorimotor pathways, and increased stiffness within the MTU have been suggested as potential explanatory mechanisms [24]. A larger stretch reflex would result in greater EMG activity, allowing greater force to be produced in SSC actions. Furthermore, considering that MTU stiffness is regulated by the amplitude and timing of the stretch reflex, a greater reliance on the short-latency stretch reflex, evident as a child matures, would allow greater stiffness upon landing, positively influencing the SSC [75]. Increased stiffness upon landing will lead to shorter ground contact times and therefore a better reutilisation of elastic energy [63]. Additionally, a stiffer MTU may elicit a greater stretch reflex in children [25], resulting in shorter breaking phases and reduced contact times.

### Rate of EMG Rise

The rate that EMG increases with contraction, represented by the initial slope of the rectified EMG curve, usually calculated over the first 30 ms of muscle activation, characterises the initial rate of muscle activation [118]. Research has demonstrated that the rate of muscle activation determines the subsequent RFD [119]. Children display significantly lower rates of EMG increase than adults [43, 96], which has been related to reduced RFD [96], suggesting that the lower rates of muscle activation negatively influences their ability to produce force rapidly. This may be due to differential motor-unit recruitment or differential rate coding of the higher-threshold type II motor units [35, 109]. Depolarising potentials are greater in amplitude for larger motor units [120]; thus, individuals who are able to recruit high-threshold motor units earlier should display a steeper rate of EMG increase, which should correspond to a heightened RFD. Theoretically, improvements in children’s ability to recruit higher-threshold motor units with maturation should translate to an escalation in the rate of EMG increase, resulting in an improved ability to produce force rapidly during the SSC.

## Trainability of Stretch-Shortening Cycle Function

A growing number of studies have examined the effects of training on SSC ability in youth, typically using a range of jump protocols to quantify indirect measures of SSC function [121]. A number of studies have demonstrated positive effects of plyometric training [122–132], traditional strength training [133–137], and combined plyometric and strength training [138, 139] on jump performance in both children and adolescents. Furthermore, recent meta-analytical data have demonstrated that various forms of resistance training can improve measures of SSC function in youth [140, 141]. From these data, plyometric training has been proven to elicit a larger overall effect on vertical jump height than interventions consisting solely of resistance training, or a combination of strength training and plyometric/speed training [141]. Research shows that the effectiveness of certain training interventions to enhance jumping performance in boys is influenced by maturation [142]. There is evidence to suggest that pre-PHV boys benefit more from plyometric training, while boys who are post-PHV respond more favourably to a combined training intervention, inclusive of both plyometric and traditional strength training exercise [142]. These maturity-dependent responses may be indicative of ‘synergistic adaptation’, which refers to the symbiotic relationship between specific adaptations of an imposed training demand and concomitant growth and maturity-related adaptations [142].

There is a significant lack of research exploring the training-induced improvements in SSC function in youth. However, research has determined the positive effect plyometric training has on motor unit recruitment, contraction velocity, excitability of soleus muscle short latency stretch reflexes and muscle activation strategies in adults [143–146]. In an adult population, research has demonstrated that an 8-week plyometric training programme results in significant increases in peak force and maximal contraction velocity in type I, type IIa and type IIb/IIx muscle fibres [146]. Similarly, a 4-week plyometric training programme was sufficient to elicit a positive training effect on the excitability of the soleus short-latency stretch reflex [145]. Plyometric training has also produced significant improvements in adductor muscle activation during the preparatory phase (150 ms prior to ground contact) during drop jump performance [144]. These studies highlight the potential effects that plyometrics have on mechanistic adaptation to muscle activation strategies in adults; however, whether the same adaptation can hold true for children and adolescents remains unclear. It may be possible to infer that the improvements in SSC function from plyometric training in children and adolescents is due to increased motor unit activation, contraction velocity, preactivation and a greater reliance on the short-latency stretch reflex, resulting in a more feed-forward SSC function.

Traditional strength training may also result in greater motor-unit activation [147, 148], resulting in positive effects on SSC function; however, this form of training is commonly linked with structural and architectural changes to the MTU. While there is a scarcity of research investigating the effects of training on muscle architecture in children and adolescents, many studies have found that resistance training alters aspects of muscle architecture in adults, specifically causing increases in muscle fascicle length [149–154], pennation angle [79, 150, 155, 156] and PCSA [79, 151]. However, it is unclear whether training will have the same effects on children and adolescents, and more research is required to understand the training-induced adaptations.

Recently, the effects of traditional strength training on the mechanical properties of the Achilles tendon have been shown to increase in stiffness following 10 weeks of twice-weekly resistance training in previously untrained pre-pubertal children [157]. This increase in tendon stiffness seems to occur due to changes to the internal properties of the tendon, as no changes to tendon CSA were found [157]. This suggests that a higher loading intensity or a greater duration of training may be required to elicit significant tendon hypertrophy. While this increase in tendon stiffness also resulted in a decreased EMD, changes to the rate of EMG rise and RFD did not occur, indicating that the magnitude of improvement in tendon stiffness (~29%), was not sufficient enough to alter these qualities [157]. Potentially, longer or more intensive training periods may yield more favourable results with respect to RFD adaptations resulting directly from greater increases in tendon stiffness.

## Conclusions

As children transition towards adulthood they demonstrate natural improvements in their ability to perform hopping and jumping tasks. Children display increases in muscle volume, fascicle length and fascicle pennation in many muscles as they mature. Additionally, tendon dimensions and mechanical properties develop with age-related body mass and force production increases, influencing its stiffness. At younger ages, the natural regulation of movement is more reactive in nature, transitioning to a more preactive control of movement as children improve their neuromuscular capacity as they age. Additionally, agonist–antagonist co-contraction may reduce as children age, stemming from a desensitisation of the Golgi tendon organs, resulting in a greater net force output. Increases in tendon stiffness, motor-unit recruitment and preactivation, and reduced co-contraction should impart a positive effect on force production characteristics and SSC function. With age and maturation, adaptations to the MTU and neuromuscular system enhance the rapid force-producing potential and result in better utilisation of the underpinning mechanisms of the SSC, resulting in an improved SSC function. Through training, SSC function seems to improve in children; however, the specific mechanisms that underpin these improvements are unclear, and further research is required to better understand the structural and neural adaptations that occur through training that lead to improved SSC function. Longitudinal studies, where key indicators of growth (size attained, rate of growth) and maturation (sexual, skeletal, age at PHV) are measured alongside SSC actions, are needed to provide more evidence into the natural development of the SSC throughout childhood and into adolescence.
